# Corrosion Evaluation of 316L Stainless Steel in CNT-Water Nanofluid: Effect of CNTs Loading

**DOI:** 10.3390/ma12101634

**Published:** 2019-05-18

**Authors:** Dana H. Abdeen, Muataz A. Atieh, Belabbes Merzougui, Walid Khalfaoui

**Affiliations:** 1 Sustainable Development Division, College of Science and Engineering, Hamad Bin Khalifa University, P.O. Box 34110 Doha, Qatar; mhussien@hbku.edu.qa (M.A.A.); bmerzougui@hbku.edu.qa (B.M.); 2 Department of Chemical Engineering, College of Engineering, Qatar University, P.O. Box 2713 Doha, Qatar; 3 Qatar Environment and Energy Research Institute (QEERI), Hamad Bin Khalifa University, P.O. Box 5825 Doha, Qatar; 4 Applied Research and Innovation Office, College of the North Atlantic Qatar, P.O. Box 24449 Doha, Qatar; walid.khalfaoui.81@gmail.com

**Keywords:** carbon nanotubes, Gum Arabic, 316L Stainless steel, potentiodynamic, corrosion, polarization parameters

## Abstract

Polarization resistance and potentiodynamic scan testing were performed on 316L stainless steel (SS) at room temperature in carbon nanotube (CNT)-water nanofluid. Different CNT loadings of 0.05, 0.1, 0.3 and 0.5 wt% were suspended in deionized water using gum arabic (GA) surfactant. Corrosion potential, Tafel constants, corrosion rates and pitting potential values indicated better corrosion performance in the presence of CNTs with respect to samples tested in GA-water solutions. According to Gibbs free energy of adsorption, CNTs were physically adsorbed into the surface of the metal, and this adsorption followed Langmuir adsorption isotherm type II. Samples tested in CNT nanofluid revealed a corrosion performance comparable to that of tap water and better than that for GA-water solutions. Among all samples tested in CNT nanofluids, the lowest corrosion rate was attained with 0.1 wt% CNT nanofluid, while the highest value was obtained with 0.5 wt% CNT nanofluid. At higher CNT concentrations, accumulated CNTs might form active anodic sites and increase the corrosion rate. SEM images for samples of higher CNT loadings were observed to have higher pit densities and diameters.

## 1. Introduction

Since the early discovery of nanofluids by Choi [1], these fluids have proven to possess unique properties due to the presence of particles that are of nanometer size (1–100 nm). Nanofluid can be formed of different fluid bases such as water, ethylene glycol (EG), oils, and so on, and nanoparticles introduced to the base fluids can be metals [2,3,4,5], oxides [4,6,7,8], carbon nanotubes (CNTs) [9,10], among others. This two-phase mixture has distinctive electrical, optical and thermal properties [11,12,13], which have allowed it to be used in many applications such as industrial heat transfer [14,15], transportation [16,17,18], biomedical [19,20] and solar applications [21,22,23]. Implementations of nanofluids in such applications have allowed energy savings, reduced emissions and design flexibility [24,25,26,27]. In addition, fine sizes of dispersed particles have conquered problems associated with the use of micro-particles, such as clogging, high operating costs and high pumping requirements [28].

CNT-water nanofluid have grabbed the attention of researchers, as it enhances the thermal properties of heat transfer fluids. The presence of nanoparticles enhances energy transport through one or more of the following four mechanisms [29,30]: (1) liquid layering on the surface of the particles, (2) particle aggregates, (3) Brownian motion and (4) Brownian motion-induced convection. CNTs have extraordinary properties that include a high modulus of elasticity [31], high aspect ratios, excellent thermal and electrical conductivity and magnetic properties [32]. Heat transport improvements in these nanofluids depend on several factors, such as CNT loading, base fluid, temperature, surfactant, and so on. A 1.14 wt% of single walled carbon nanotubes (SWCNT) in water-based nanofluid showed a 19.4% enhancement in thermal conductivity of the base fluid, water [33]. CNT-water nanofluids have been recorded to achieve 38% and 34% thermal conductivity enhancements when the nanofluids were prepared with sodium dodecyl sulfate (SDS) and cetyltrimethylammonium bromide (CTAB) surfactants, respectively [34,35]. In another study, gum arabic (GA) was proven to provide the best thermal conductivity amongst sodium dodecylbenzene sulfonate (SDBS) and SDS surfactants, with an optimum sonication time of 40 min and at 45 °C [36]. Furthermore, thermal conductivity was shown to increase with increasing CNT loading and temperature. A multiwalled carbon nanotube (MWCNT) with 0.25 wt% GA suspended in water achieved 18% and 37% improvements in the thermal conductivity of 0.1 and 0.5 wt% CNT-water nanofluids, respectively [37]. In addition, a maximum thermal conductivity enhancement of 35.9% was recorded when 1 wt% CNT-water nanofluid was prepared with CTAB surfactant at 60 °C [38].

A lot of research work has been conducted to check the heat transport properties of nanofluids for utilization as a heat transfer fluid. However, less effort was placed on understand the corrosion effect of nanoparticles on metallic substrate [39]. Introduction of nanoparticles to the base fluids alters their thermo-physical properties, which might cause a secondary mechanical (abrasive, erosion) or chemical (corrosion) effect [40]. In addition, the presence of some chemical surfactants like GA might provide an inhibition act [41,42,43,44], and might affect the electrochemical behavior of metals exposed to such solutions of nanoparticles and surfactant.

Studying the corrosion of exposed surfaces to the nanofluid is essential not only from an economic perspective, but also for safety and health. Corrosion is the deterioration of metal due to a reaction with corrosive elements [45]. Corrosion can cause problems of degradation, failure and serious accidents in many industrial plants and domestic systems. Economic influence due to corrosion can include loss of materials, repair and maintenance cost, decrease in efficiency, and so on. Corrosion can also cause fires and explosions, in addition to other health impacts of personal injuries and pollution [46]. Globally, the impact of corrosion are estimated to be $2.5 trillion USD, which counts for 3.4% of the global gross domestic product (GDP) [47].

Until now, no comprehensive research work has been carried out to understand corrosion behavior in the presence of heat transfer fluids. Most of the research work has focused on the investigation of heat transfer properties along with corrosion properties [40,48], while others have focused on erosion properties [49,50] or the effects of some experimental conditions [51]. For example, Baghalha and Kamal Ahmadi found that increasing the concentration of SDS surfactant caused an increase in the corrosion of copper, while stirring did not change the corrosion behavior of tested samples. In addition, it has been observed that exposing copper samples to CNT-water nanofluid reported lower current densities compared with samples tested in pure water [51]. Corrosion of different metals exposed to CNTs in water with suspended carboxylate additives were examined through an immersion test for 14 days at 80 °C. It was concluded that this nanofluid with different CNT loadings and different additives was suitable to use in automotive environments, and that the presence of CNTs did not affect the corrosion properties of the water that contained a carboxylate additive [48]. On the other hand, the corrosion rates of copper, stainless steel (SS) and aluminum samples exposed to CNT-water and CNT-ethylene glycol (EG) nanofluids increased with the temperature. At a fixed temperature, copper and aluminum had the highest and lowest corrosion rate values, respectively [40]. Celata et al. examined the erosion effect of metallic samples exposed to different nanofluids using a Hydraulic Experiment on Thermo-mechanical of Nanofluids (HETNA) experimental rig. The measured thickness loss obtained was not identified, whether it was due to an erosion or to corrosion effect [49]. Continuing from the work of Celata et al., Bubbico et al. changed the pH in the presence of the same nanofluids, and concluded that the measured material loss was mainly due to chemical corrosion, rather than from mechanical erosion [50].

As CNT-water nanofluid has the potential to be used in many applications, it is important to keep safety considerations in regard to corrosion issues. Very limited work has been conducted in this regard, and none of it has been comprehensive or standardized. This paper measures the corrosion parameters of 316L stainless steel exposed to a potential heat transfer fluid, CNT-water nanofluid. Three corrosion tests were conducted in series: an open circuit test, a polarization resistance test and potentiodynamic scans. Examinations were done at different CNT loadings of 0.05, 0.1, 0.3 and 0.5 wt% CNTs that were homogenously dispersed in deionized water using GA surfactant. Corrosion potential, Tafel constants, corrosion rate and pitting potential were measured and analyzed. For further understating of the corrosion effects of CNT addition, the same tests were conducted for GA-water solutions using the same GA concentrations as in the preparation of the CNT nanofluids. In addition, the same tests were performed in deionized water, tap water and 3.5 wt% NaCl solutions. The adsorption process of CNTs and/or GA molecules were examined by calculating Gibbs free energy of adsorption and finding the adsorption isotherm. In the end, these data were gathered to propose a mechanism for corrosion/inhibition of steel in presence of CNTs.

## 2. Experimental Work

### 2.1. Nanofluid Synthesis

CNT-water-based nanofluid was prepared using a two-step method, where the MWCNTs are synthesized first and then dispersed into deionized water. Multiwalled CNTs were purchased from Cheap Tubes. They were manufactured through a catalytic chemical vapor deposition (CCVD) process and refined to a purity of >95% using a concentrated acid chemistry method. Specifications for the MWCNTs used are shown in Table 1.

For synthesizing a homogenous nanofluid, gum arabic (GA) from Sigma-Aldrich was used as a surfactant, as it proved to sustain high temperatures without foaming, unlike SDBS surfactant [52]. A weight ratio of 1:3 (CNT:GA surfactant) was used. The solutions were then sonicated by an ultrasonication probe (vibra-cell) from Sonics & Materials. The sonication parameters were fixed at 20 kHz and 450 watts for 1 h at room temperature. The prepared CNT-water-based nanofluids were homogenous for a long time, with no visual sedimentation observed after one month. Finally, for comparison purposes, samples were tested in deionized water, GA-water solutions (of the same concentrations used in CNT-water nanofluids), tap water and 3.5 wt% NaCl solutions. The last two solutions were chosen because CNT-water nanofluid has the potential to replace fresh water and seawater when used for cooling/heating purposes. Tap water used was analyzed in Gulf Laboratories CO. W.L.L., with the composition listed in Table 2 [53].

### 2.2. 316L Stainless Steel Samples Preparation

The 316L stainless steel (SS) samples (composition shown in Table 3) were purchased from the company Metals Samples. Samples were processed by drawing and chamfering, then roller polishing into cylindrical rods of 1/4” (6.35 mm) diameter X 2 1/2” long (63.50 mm), with no threads or slots. Purchased samples were prepared into a working electrode by having the rods cut, glued into a copper wire and mounted in an epoxy resin on all sides except the surface to be tested. The final step of sample preparation was the wet polishing of the mounted samples with 1200-grit silicon carbide paper. The AutoMet Grinder Polisher (Buehler AutoMet 250), was used for this step.

### 2.3. Corrosion Testing

Studying the corrosion behavior was conducted by running three corrosion tests in series for each sample, including an open circuit potential test (OCP), a polarization resistance test and a potentiodynamic scan test. Experiments were conducted using a 600 Potentiostat and EuroCell kit from Gamry Instruments. Experimental procedures implemented the ASTM G59-97 and ASTM G5-14 standards to perform polarization resistance testing and potentiodynamic scanning, respectively [54,55]. The mounted 316L SS samples were wet-polished with 1200-grit silicon carbide paper and washed with ethanol, then with deionized water. A graphite rod was used as a counter/auxiliary electrode, and a saturated calomel electrode (SCE) was used as a reference electrode. To avoid any shorting between working electrodes and counter electrodes due to the presence of carbon nanotubes, the graphite rod was placed inside a nafion membrane that allowed chemical (but not physical) contact with the test solution. Samples were immersed in the test solution for 10 h to allow the surface of the metal to settle and form an oxide layer, and to establish equilibrium potential, that is; the open circuit potential (OCP). This test conducted for the polarization resistance test was run at a scan rate of 0.6 V/h (0.167 mV/s), and under a potential range of 0.25 V above and below the E_OCP_ that was obtained from the OCP test. The potentiodynamic test was performed at the same scan rate, and was run from −0.25 V below E_OCP_ to 1.5 V_SCE_. After potentiodynamic scanning, samples were removed from the cell and allowed to air-dry for SEM observation. Mounted 316L SS samples were exposed to four concentrations (0.05, 0.1, 0.3 and 0.5 wt%) of CNT-water nanofluids. Three replicates for each solution were tested to ensure consistency of the results. All experiments in this part were performed at room temperature (22 °C), and the measured pH of nanofluids had an average value of 5.6.

## 3. Discussion of Results

### 3.1. Potentiodynamic Scans

#### 3.1.1. Potentiodynamic Scans in the Presence of CNT-Water Nanofluids

**Figure 1:**
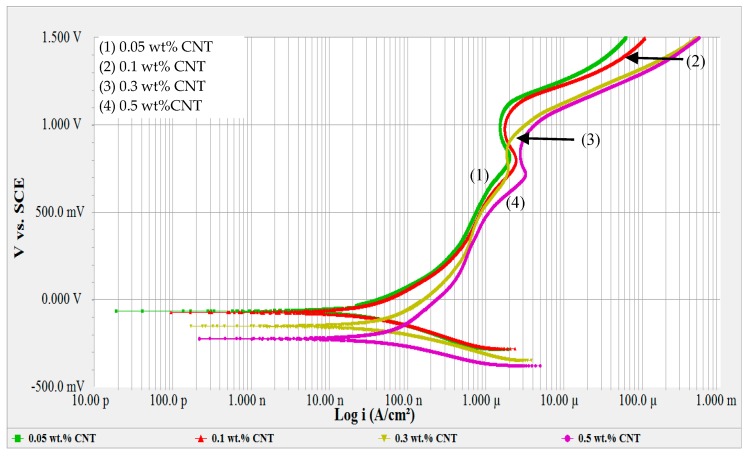
Potentiodynamic scans for 316L stainless steel (SS) exposed to 0.05, 0.1, 0.3 and 0.5 wt% CNT-water nanofluids at room temperature.

After immersing the samples for 10 h in an OCP test, a potentiodynamic test was conducted. The potentiodynamic curves were constructed and the results are illustrated in Figure 1. Potentiodynamic curves are plotted for log current density versus potential [45]. These curves represent the anodic and cathodic reactions occurring simultaneously, but some of these reactions will proceed at relatively higher rates, depending on the applied potential. Few turning points were observed, which was due to the change in the kind of oxides formed on the surface of the metal [54]. Figure 1 shows the potentiodynamic curves for 316L stainless steel exposed to CNT-water nanofluid with 0.05, 0.1, 0.3 and 0.5 wt% of CNT. Lower CNT loadings of 0.05 and 0.1 wt% were shown to have almost the exact behavior in terms of passivation region and oxide film formation. On the other hand, increasing the CNT loadings to 0.3 and 0.5 wt% seemed to affect that corrosion behavior, as was seen from their potentiodynamic curves. In general, the stainless steel tested in the CNT-water nanofluids revealed a passive–active–transpassive–active behavior, as was observed from the change of current density with the increase in potential in their polarization curves.

Each regime in the curve represented an electrochemical reaction occurring on the surface of the sample. In the current neutral aerated environment, a series of anodic and cathodic reactions can occur at the surface of the electrode that can include cathodic oxygen reduction (H_2_O + ½ O_2(g)_ + 2 e^−^ → 2OH^−^), anodic iron dissolution (Fe → Fe^+2^ + 2e^−^) and hydrogen evolution reaction (2H^+^ + 2 e^−^ → H_2(g)_). At a lower potential, cathodic reactions are expected to proceed at higher rates than the anodic reactions, according to the Eh-pH diagram, particularly with neutral or alkaline media. At higher potentials, anodic reactions proceed at higher rates, causing metal dissolution, while at the same time oxides are formed on the surface of the metal.

With the oxidation of the surface of the metal, a film starts to develop on the surface that consists of different oxides such as iron hydroxide (Fe (OH)_2_), hematite (αFe_2_O_3_) and magnetite (Fe_3_O_4_). Some of these oxides are formed according to reactions (1)–(4) reported in the literature [55]. In addition, chromium oxides in aqueous solutions to produce Cr_2_O_3_ according to reaction (5).

(1) $$\mathrm{Fe}+H_{2}O\;\;\overset{\leftarrow }{\to }\mathrm{Fe}O_{\left(s\right)}+2H^{+}{}_{\left(\mathrm{aq}\right)}+2\;e^{-}$$

(2) $$\mathrm{Fe}+2\;H_{2}O\;\;\to \;\mathrm{Fe}{\left(\mathrm{OH}\right)}_{2}{}_{\left(s\right)}+\;\;2H^{+}{}_{\left(\mathrm{aq}\right)}+2\;e^{-}$$

(3) $$3\;\mathrm{FeO}+\;H_{2}O\;\;\to \;{\mathrm{Fe}}_{3}O_{4}{}_{\left(s\right)}+\;2H^{+}{}_{\left(\mathrm{aq}\right)}+2\;e^{-}$$

(4) $$2\;{\mathrm{Fe}}_{3}O_{4}{}_{\left(s\right)}+\;H_{2}O\;\;\to \;3{\left(\gamma -Fe_{2}O_{3}\right)}_{\left(s\right)}+\;\;2H^{+}{}_{\left(\mathrm{aq}\right)}+2\;e^{-}$$

(5) $$4\;{\mathrm{Cr}}_{\left(s\right)\;}+\;3\;O_{2}\;\to 2\;{\mathrm{Cr}}_{2}O_{3}{}_{\left(s\right)}$$

These oxides and hydroxides precipitate on the surface of the metal and cause the metal to transfer from an active state to a passive state. It was reported that a bi-layer film formed on the surface of the 316L SS consisting of an inner and deeper chromium oxide layer, and that another outer layer consisting of iron oxides formed on the surface of the steel [56,57]. This barrier film formed on the surface restricted electrical conductivity and reduced the current density.

Formation of such oxides allowed the sample to passivate, which caused the current density to have a minimum change in a specific potential regime. Few turning points were detected within the passive regime for all curves. These turnings are an indication of different oxide formations [54], as each oxide is found to be stable and to dominate in a specific potential range. The passivation region started with all nanofluids almost at the same passivation potential (E_pp_) between 0.20–0.30 V_SCE_, and with a passivation current density ranged between 0.5 and 1.0 µA/cm^2^. The thickness of the film is expected to increase with increasing potential according to the availability and the activity of the hydroxide and ferrous ions on the metal–electrolyte interface [58,59]. Such film formations are supposed to bring clear straight passive regions in the polarization curves, as observed by Lothongkum [60] when 316L SS was tested in aqueous solutions without any chloride ions. Curves (1) and (2) of Figure 1 show the potentiodynamic scans for 0.05 and 0.1 wt% CNT-water nanofluids, respectively. For these two nanofluids, passivation appeared in the potential window of 0.30–1.15 V_SCE_ without a significant shift in E_corr_ between the two CNT loadings. However, higher CNT concentrations of 0.3 and 0.5 wt% (curves (3) and (4) of Figure 1, respectively) passivated in the potential window of 0.20–0.95 V_SCE_ with less repassivation behavior, especially for the 0.3% CNT nanofluid. In addition, increasing CNT loading showed a higher shift of E_corr_ towards more negative values. A shift towards more negative values of E_corr_ indicates a higher susceptibility of the material to corrode, as it can be attacked at lower potentials. Such passivation behavior indicates that the presence of CNTs and GA surfactant influenced the film formation on the surface of the metal, especially for higher CNT loadings.

#### 3.1.2. Potentiodynamic Scans for GA-Water, Tap Water and NaCl Solutions

For a better understanding of the real effect of CNTs on the corrosion of the tested samples, potentiodynamic scans in GA-water solutions were also performed at different concentrations of GA. Corrosion behavior in presence of CNT-water nanofluids was also compared with conventional heat transfer fluids such as tap water and 3.5 wt% NaCl solution, with the latter solution having the same sodium chloride composition as sea water that is commonly used for cooling purposes.

**Figure 2:**
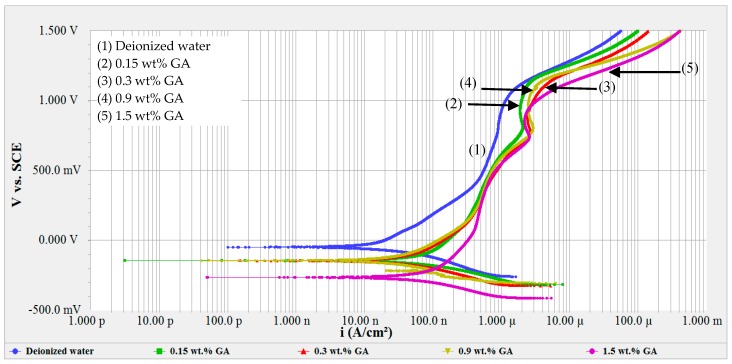
Potentiodynamic scans for 316L stainless steel exposed to GA-water solutions of 0.15, 0.3, 0.9 and 1.5 wt% gum arabic (GA).

Furthermore, corrosion testing in GA solutions was performed in order to examine the effects of GA in CNT-water nanofluids, since GA is considered as a natural corrosion inhibitor [41,61]. The same weight of GA used in CNT-water nanofluid was tested in this section, but without CNTs. GA was introduced in the nanofluids with a 3:1 ratio (GA:CNT). Similarly, the same concentrations of GA were sonicated in the deionized water and used for testing. The tested GA-water solutions had GA weight percentages of 0.15%, 0.3%, 0.9% and 1.5%, which corresponded to the same amounts of GA present in CNT-water nanofluids of 0.05, 0.1, 0.3 and 0.5 wt%, respectively. Figure 2 shows the potentiodynamic scans for 316L SS tested in different GA-water solutions.

Polarization curves of GA-water solutions shown in Figure 2 presented almost the same passivation behavior found in CNT-water nanofluids (Figure 1). In both Figures, active–passive–transpassive–active behavior was noted and the same turnings were observed. This is an indication of different oxide formations on the surface as the result of the presence of GA, regardless of whether CNTs were present in the solution or not. However, CNT affected the corrosion rate, corrosion potential and Tafel constants, as will be discussed later in detail. The passive region starts at around 0.2 V_SCE_, passes through a transpassive state and secondary passivity at 0.8 V_SCE_, then returns to the active state at around 1.15 V_SCE_. The passivation current was observed to be in the range of 0.5–1.1 µA/cm^2^.

**Figure 3:**
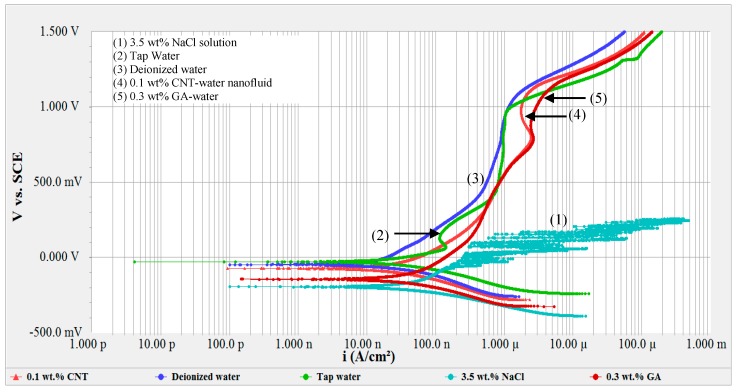
Potentiodynamic scans of 316L stainless steel in different solutions.

Figure 3 shows the potentiodynamic curves of 316L SS tested in different solutions. Comparing the passivity region between all solutions, it is evident that the steel did not show a passive behavior in the presence of NaCl solution due to the presence of aggressive chloride ions, which is to be expected. On the other hand, the active–passive region of tap water and deionized water was different from that of the nanofluid and GA solutions. Tap water and deionized water showed a passive behavior in the region of 0.43–0.97 V_SCE_, unlike the CNT-water nanofluid and the GA-water solutions. Tap water had clear turnings of the curve at around 0.07 and 0.13 V_SCE_ due to the formation of stable iron and chromium oxides on the surface. A firm passive film was noticed in the potential range of 0.49 and 0.97 V_SCE_, unlike the CNT-water nanofluid and the GA-water solutions. The tap water had clear turnings on the curve at around 0.067 and 0.126 V_SCE_ due to the formation of stable oxides on the surface. A firm passive film was noticed in the potential range of 0.4 and 1.0 V_SCE_. Such behavior for tap water is consistent with what was reported elsewhere [60,62]. However, the passivation region for the GA-water solution and CNT-water nanofluids did not have clear turnings, nor did they have a sharp reduction in current density. This could be due to the existence of the surfactant GA and CNTs that were adsorbed on the surface of the metal and added extra resistance in the bulk electrolyte. Presence of these species covered parts of the sample surface and affected oxide formation on the tested surface.

### 3.2. Inhibiting Effect of CNT-Water Nanofluid

#### 3.2.1. Corrosion Potential (E_corr_)

Table 4 shows the corrosion potential values obtained after conducting the OCP and polarization resistance tests followed by potentiodynamic scans, for 316L stainless steel tested in different solutions at room temperature. Corrosion potential (E_corr_) is essential for understanding the mechanisms of corrosion inhibition with the addition of species to electrolyte solutions. E_corr_ values are obtained from the polarization curve with the extrapolation of the Tafel lines. Comparing E_corr_ values of solutions with and without CNTs can help identify the dominant effect of corrosion inhibition due to the addition of the CNTs. Having no change or small change (less than 85 mV) in E_corr_ with the addition of a component is an indication that this component affected both reactions. A change or a shift in the E_corr_ value signifies that the added species affected one of the reactions more than the other, depending on the direction of shifting [63,64,65].

In this neutral solution, the anodic reaction is the dissolution of 316L stainless steel ions into the solution from the metal surface. Cathodic reactions are overall associated with the reduction of metal cations and water to deposit metal and form hydrogen gas, respectively. A compound can be classified as anodic type or cathodic type if the change in E_corr_ values is higher than 85 mV [65,66]. Since the displacement for E_corr_ between GA-water solutions and the corresponding E_corr_ values for CNT nanofluids was less than 85 mV, the CNT in the solution was affecting both anodic and cathodic reactions. The presence of CNTs and GA in deionized water solution reduces the dissolution of 316L SS and retards the hydrogen evolution and hydroxyl formation reactions.

The addition of CNT affected both reactions, but there was a dominant effect towards one of the cathodic or anodic reactions. A positive displacement of E_corr_ values was observed for CNT-water nanofluids compared with their corresponding GA-deionized water solutions. The highest positive displacement (59 mV) was observed in 0.3 wt% CNT-water nanofluid, compared with that for the corresponding 0.9 wt% GA-deionized water solution. This signifies that CNTs were more affective towards the anodic reaction; that is, CNTs occupied anodic sites and decreased metal dissolution at a higher rate than they decreased cathodic hydrogen evolution reaction. With the positive shift of E_corr_ values, decreasing anodic reaction might have occurred as a result of the adsorption of GA and CNTs on the surface of the metal, which led to an enhancement of the stability of the metal oxide layer and the CNTs layer [67]. A small displacement of E_corr_ was also noted with the addition of CNTs and SDS surfactant to deionized water, but it was negative displacement [68].

**Figure 4:**
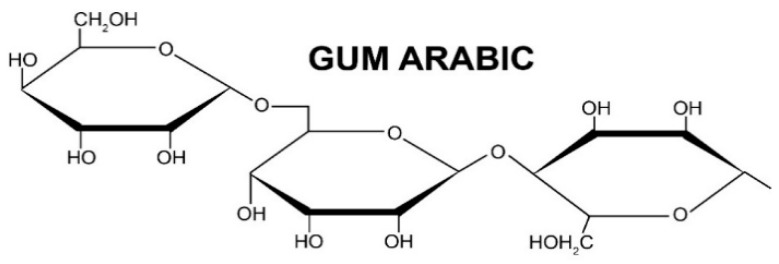
Molecular structure of gum arabic [74].

GA is a natural inhibitor that is soluble in water and contains a mixture of polysaccharides, sucrose, oligosaccharides, arabinogalactan and glucoproteins [69,70]. GA plays a great role in providing the needed corrosion inhibition in the surface of metallic substrates. It is an anionic surfactant with a hydrophobic tail and hydrophilic head, and it is connected to metal surfaces though its hydrophilic head, which is attracted to the polar negatively charged surface of the oxides [61]. GA contains constituents in the form of hetero atoms such as oxygen, which will provide active adsorption sites [71]. With the structure of GA shown in Figure 4 [71], the interaction happens between the ferrous ions and the oxygen atom in the backbone of the polymer, which provides the right coordination for bonding [72]. On the other hand, CNTs are hydrophobic in nature, hence their surfaces need to be modified by attachment with some groups in order to overcome their weak interfacial interactions with aqueous solutions and allow their dispersion in such solutions [73]. GA is added to CNT-water solution as a surfactant to allow homogenous dispersion of the hydrophobic CNT in water.

Adsorption on the surface of the metal is recognized as the mechanism to explain the retardation action of organic inhibitors such as GA [64,75]. Adsorption of inhibitors usually decreases the corrosion rate in two ways [63]: (1) by decreasing the available surface area for reactions, which is called the geometry blocking effect; and (2) by changing the activation energy needed for cathodic/anodic reactions. Determining the dominant effect is difficult, as more than one parameter can influence the result. Theoretically, no change in the E_corr_ for the solution after the addition of the inhibitor indicates that the geometry blocking effect is stronger than the activation energy effect [63,64]. E_corr_ values for all GA-water solutions had a small shift towards more negative values compared with those for deionized water, highlighting the blocking’s role in the inhibition of GA-only solutions in addition to the activation energy effect. Furthermore, the small positive displacement observed for E_corr_ values of CNT nanofluids also indicated the small role of the surface energy effect in CNT adsorption, and hence in corrosion inhibition. It can be said that the blocking effect of CNTs had a major contribution to corrosion inhibition, without ignoring the surface energy effect.

#### 3.2.2. Polarization Parameters

Table 5 shows some corrosion parameters for 316L SS tested in different solutions. Polarization parameters of anodic (β_a_) and cathodic (β_c_) Tafel constants, corrosion current density (i_corr_), polarization resistance (R_p_), pitting potential (E_pit_) and corrosion rate were gathered after conducting three tests in series: OCP, polarization resistance test and the potentiodynamic scan.

Potentiodynamic scans of CNT-water nanofluids and of the GA-water solutions are shown in Figure 1 and Figure 2, respectively. A passive–active–transpassive–active behavior was observed, with or without CNTs. Typically, adding an inhibitor affects one of the redox reactions, or both reactions. An inhibitor is supposed to decrease the anodic Tafel slope and slightly increase the OCP after the immersion. No change or negligible change in the cathodic Tafel slope indicates that the hydrogen evolution is diminished by the surface blocking effect of the inhibitor [63].

Tafel constant values β_a_ and β_c_ were found to change with the addition of GA to deionized water, indicating that GA affected both reactions and confirming that GA is a mixed-type inhibitor [43,75,76]. Addition of CNTs to the GA-water solution decreased both β_a_ and β_c_ values; however, the change in β_a_ was higher. This confirms the same finding as the slight shift of E_corr_ of a higher influence on the anodic reaction. At the same time, the decrease in β_c_ was not significant with the addition of CNTs, indicating the blocking effect of CNT in diminishing the hydrogen evaluation reaction. Hence, it can be acknowledged that presence of CNT inhibited the corrosion of the stainless steel by blocking parts of the exposed surface, with some influence on the surface energy of the steel.

**Figure 5:**
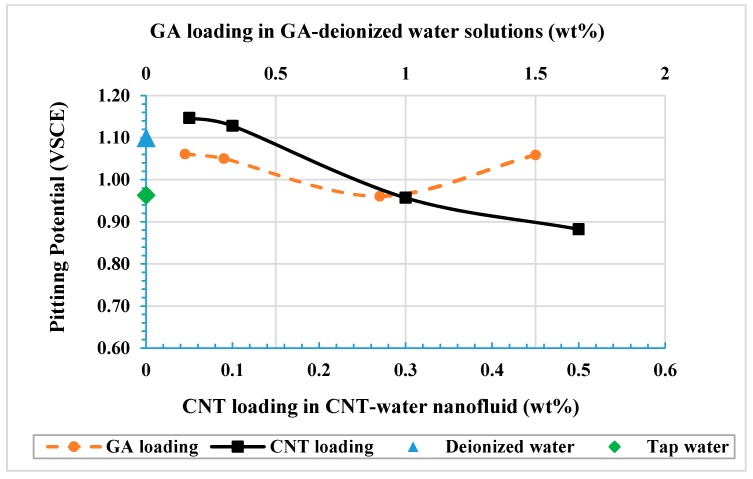
Effect of different loadings of CNT-water nanofluids and of GA-water solutions on the pitting potential of 316L stainless steel.

Pitting potential (E_pit_) is also a corrosion parameter that can give insight into understanding the susceptibility of the tested sample to corrosion in a specific condition. E_pit_ is usually obtained from the potentiodynamic scan curve where an abrupt increase in current density is detected. In the current solutions, an abrupt increase in current density was not noticeable, so E_pit_ was considered as the potential at which the current density increased at a higher rate and kept on increasing without any passivation state (re-passivation is where there is retarding in current density value). However, it is important to mention that an increase in the anodic current does not entirely represent pitting; it can also result from oxygen evolution (water oxidation) reaction, although for this instance we can assume that pitting was more dominant. Therefore, the average pitting potential values (E_pit_) can be estimated from the curves, and are listed in Table 5. It is noticed from curve (1) in Figure 5 that the pitting potential in the NaCl solution was the lowest compared to all other solutions due to the presence of aggressive chloride ions. When tested in NaCl solution, pitting initiated at an average potential of 0.187 V_SCE_. This value is lower than the 0.286 V_SCE_ obtained by Saadi et al. when they tested 316L in 3.5 wt% NaCl solution [56]. On the other hand, Table 5 shows that lower concentrations of CNT nanofluids (0.05 and 0.1 wt%) had the highest E_pit_ values, which were higher than those for deionized water and tap water. This was due to the inhibition effect and adsorption of the GA and CNT on the surface of the metal, which enabled the surface of the metal to sustain higher potentials. Higher CNT concentrations are associated with lower pitting potential values and with higher corrosion rates. Such increases in metal dissolution with higher CNT concentrations might be due to the formation of active anodic sites in the stagnant regions between the accumulated CNTs and the surface of the metal. It might be also because of the desorption of the CNTs from the surface with increased CNT concentration [64].

Another parameter to investigate is polarization resistance (R_p_). Table 5 shows that lower R_p_ is associated with higher corrosion rates. Testing in NaCl solution resulted in a lowest resistance of 839 kohm, while deionized water had the highest R_p_ value of 7994 kohm. In addition, testing in GA-water had R_p_ values in the range of 2634–3667 kohm, while nanofluids and tap water had R_p_ values in the range of 3777–5950 kohm. Presence of CNTs in nanofluids resulted in higher resistance in the electrolyte solution. Among all concentrations of CNT nanofluids, 0.1 wt% resulted in the highest polarization resistance, while further increases in CNT concentration led to a decrease in that resistance. In addition to solution resistance and charge transfer resistance, CNTs formed a thin film carbon coating on the surface of the metal that created extra resistance. However, at higher concentrations of CNTs, anodic reaction accelerated at a higher rate and increased the corrosion rate. This is in agreement with the increase in β_a_ values obtained for the mentioned two highest CNT concentrations.

Table 5 shows the corrosion rate values of all tested solutions. As can be seen from the presented average values, the highest corrosion rate was obtained with the NaCl solution, with a value of 44.34 mmpy (milli mil per year, equivalent to 0.0011 mm/yr) and a current density of 0.1488 µA/cm^2^. Such a high corrosion rate was associated with the highest i_corr_ and lowest R_p_ values among all other tested solutions. Testing samples using deionized water resulted in lower corrosion rates than in tap water, which was as expected since the latter solution contained more ionic species than deionized water. For the same reason, the corrosion rate for GA in deionized water was higher than the corrosion rates of deionized and tap water, despite the fact that GA is an inhibitor. Furthermore, adding CNTs to the GA solution decreased the corrosion rate at all concentrations of CNT nanofluids. A minimum corrosion rate of 6.43 mmpy was obtained with 0.1 wt% CNTs nanofluid. Increasing CNTs loading higher than 0.1 wt% caused the corrosion rate to increase with increasing CNTs concentration. This indicates that introducing CNTs to the GA-water solution increased the resistance of the bulk solution and lowered the corrosion rate due to the adsorption of CNTs and the GA on the metal surface. The presence of such species covered part of the metal’s exposed surface and decreased the anodic reaction. However, if the coverage was not uniform it could accelerate the corrosion by providing areas of different potential values within the metal surface.

Previous work related to the present study was limited in scope and volume. Rashmi et al. tested stainless steel in 0.1 wt% CNT-water nanofluid and achieved a corrosion rate of 2.48*10^−5^ mm/yr [40], which is relatively close to our value of 1.63*10^−4^ mm/yr. In addition, an immersion test was used to measure the corrosion rate of 316L stainless steel in de-mineralized water and in distilled water. It was found that the corrosion rate in both de-mineralized water and distilled water were around 0.0608 and 0.0476 mm/yr, respectively [77]. Both values are higher than the corrosion rate obtained in the current research (equivalent to 1.15 × 10^−4^ mm/yr), which might have been due to the method followed in measuring the corrosion rate. An immersion test method is usually not recommended when the material is expected to have low corrosion rates in the tested media [78]. Furthermore, Rashidi et al. measured the corrosion rate of carbon steel in 0.1 wt% MWCNT dispersed in distilled water and found corrosion rate values of 3.3402 and 1.643 mpy for SDBS and SDS surfactants used to stabilize the CNTs, respectively [68].

**Figure 6:**
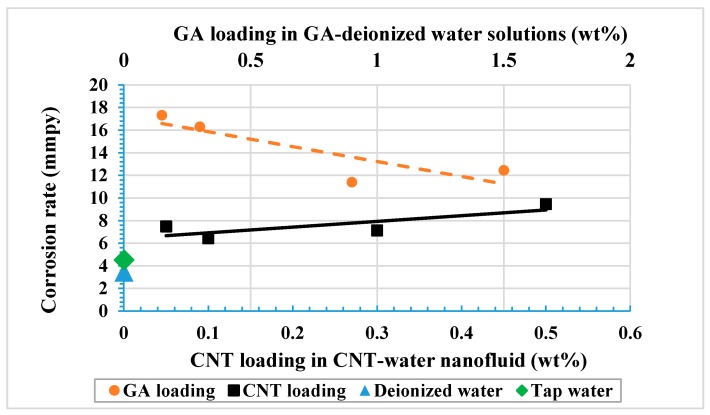
Corrosion rate for tested CNT-water nanofluids and for GA-water solutions.

Figure 6 shows corrosion rate values for samples tested in CNT nanofluids and for GA-water solutions. A linear relation was proposed between the corrosion rate and the CNT loading that appeared to be a positive relation. On the other hand, GA was revealed to have a negative linear relation as the corrosion rate decreased with increasing GA concentrations. Similar results of decreasing corrosion rates were found with increasing GA concentrations in potable water [76] and in an acidic medium [42,75]. Furthermore, other surfactants such as SDS and SDBS used to disperse CNTs in distilled water did not show an inhibition effect. Both surfactants were found to have higher corrosion rates compared to those for distilled water [68].

The decrease in corrosion rate due to the presence of CNT can be evaluated by calculating the inhibition efficiency (*IE*). Inhibition efficiency is the percentage of corrosion rate decrease with the presence of CNT with respect to the corrosion rate value in CNT-free solution. Inhibition efficiency (*IE*%) can be calculated as in Equation (6):

(6) $$IE\left(\%\right)=\frac{CR\;\left(CNTs-free\;solution\right)\;-\;CR\;\left(CNTs\;nanofluid\right)}{CR\;\left(CNTs-free\;solution\right)\;}\times 100\%$$

where *CR* (CNT free solution) is the corrosion rate of 316L stainless steel samples tested in GA-water solution, and *CR* (CNT nanofluid) is the corrosion rate of the same steel tested in CNT-water nanofluid. The inhibitor’s surface coverage (*θ*) can also be calculated as the fraction of the inhibition efficiency (IE%/100), as listed in Table 6. A highest inhibition efficiency of 61% was obtained with 0.1 wt% CNT nanofluid, while a lowest inhibition of 24% with 0.5 wt% CNT nanofluid. Comparing with the inhibition effect of GA in the literature, it was found that a maximum inhibition of 96.8% was achieved with the adsorption of acacia gum (600 ppm) on mild steel where the testing media were potable water and at 80 °C [76].

#### 3.2.3. Adsorption Isotherm

In order to evaluate the adsorption process of CNTs and the surfactant GA on the surface of the metal surface, it is important to determine the adsorption isotherm that best fits our data. Adsorption isotherms provide insights into the interactions between adsorption molecules and the surface by plotting the surface coverage of the adsorbed inhibitor (*θ*) versus the equilibrium concentration of the inhibitor (*C*). Different adsorption isotherms were checked for their applicability on the results using Langmuir, modified Langmuir, Langmuir-Freundlich, Freundlich, Temkin, Flory–Huggins and Frumkin equations. It was found that only the modified Langmuir type I, modified Langmuir type II and Flory–Huggins equations were applicable to our experimental data. Adsorption isotherms were obtained according to Equations (7)–(9) [79]:

Modified Langmuir isotherm type I:

(7) $$\frac{C}{\theta }=nC+\;\frac{n}{K_{ads}}$$

Modified Langmuir isotherm type II:

(8) $$\frac{\theta }{\theta -1}\;=nK_{ads}C$$

Flory–Huggins isotherm:

(9) $$\frac{C}{\theta }=K_{ads}{\left(1-\theta \right)}^{x}$$

where *C* is the equilibrium inhibitor concentration in g/L, *θ* is the surface coverage of the adsorbed inhibitor, *K_ads_* is the adsorption equilibrium constant and *n* and *x* are parameters associated with the number of displaced water molecules per active site on the surface of the metal and consequently the number of adsorbed inhibitor molecules.

**Figure 7:**
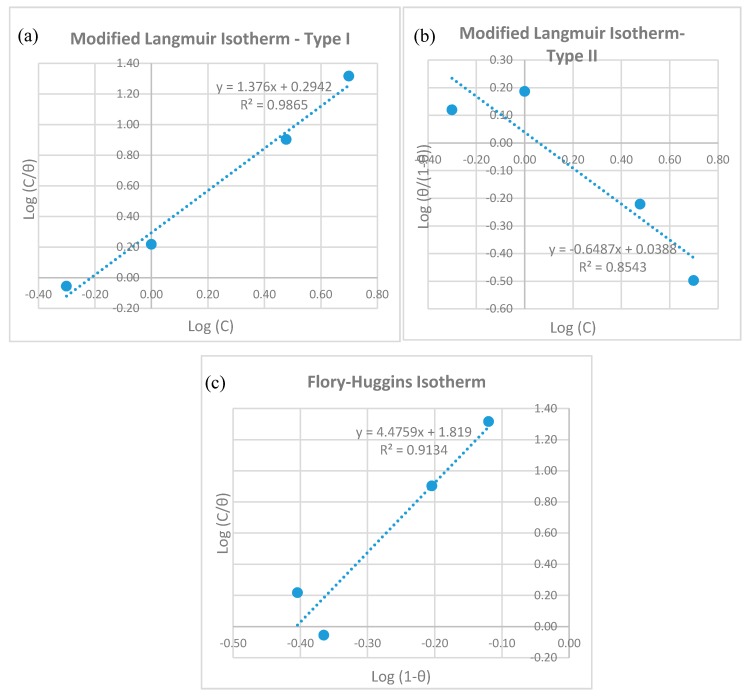
Adsorption isotherm plots for CNTs and gum arabic adsorbed on 316L stainless steel after potentiodynamic testing at 22 °C.

To determine which model would best fit the surface coverage of the adsorbed CNTs and GA species, the linearized forms of these equations are plotted in Figure 7. The modified Langmuir type I isotherm showed the best representation of the data, as it had a regression constant value (R^2^) of 0.9865. The Flory–Huggins isotherm matched the data better than modified Langmuir type II, with both having R^2^ values of 0.9134 and 0.8543, respectively. The parameter *n* was obtained from the slope of the modified Langmuir type I correlation, and had a value of 1.38, indicating that there was more than one CNT and/or GA adsorbed at a particular active site on the surface. In addition, having an *n* value in that model that was not equal to unity signifies that there were interactions between adsorbed CNTs and GA species [79]. The adsorption constant *K_ads_* was also obtained from the intercept of the same correlation, and had a value of 0.61 L/g. The value of *K_ads_* represents how strongly and efficiently the adsorbed species are attached to the surface [79,80]. A low value of *K_ads_* is expected with physically adsorbed molecules.

Finding the value of *K_ads_* can also provide the amount of adsorption free energy (ΔG°_ads_) according to Equation (10):

(10) $$∆G_{ads}^{{}^{\circ}}=\;-R\;T\;\ln\left(K_{ads}\;C_{solvent}\right)$$

where *R* is the gas constant, *T* is absolute temperature and *C_solvent_* is the concentration of solvent (equal to 999 g/L for water). A negative value of Δ*G°_ads_* indicates that the adsorption of the surfactant is spontaneous and that its interaction with the surface is strong. Having Δ*G°_ads_* that is more positive than −20 kJ/mol indicates that the adsorption is a physisorption process. On the other hand, if the process is more negative than −40 kJ/mol, then the adsorption will be classified as chemisorption, as it involves charge sharing or transfer to form bonds between surfactant and solid surface [61,64,65]. According to our data, Δ*G°_ads_* had a value of −15.73 kJ/mol, indicating it was a spontaneous adsorption. Since the value of Δ*G°_ads_* was more positive than −20 kJ/mol, then CNTs and GA were adsorbed physically to the surface of the metal. Electrostatic interactions occurred between the negatively charged surface of the metal and the adsorbed molecules, rather than chemical bonding. As the mechanism of adsorption was a physical one, the process could be reversed, and a desorption of the molecules might happen, especially at higher temperatures or concentrations. A physical adsorption of GA on the surface of steel was obtained in 0.1 M H_2_SO_4_ solution [71], in 0.1 M HCl solution [81] and in potable water [76].

### 3.3. Scanning Electron Microscope (SEM) Observations

**Figure 8:**
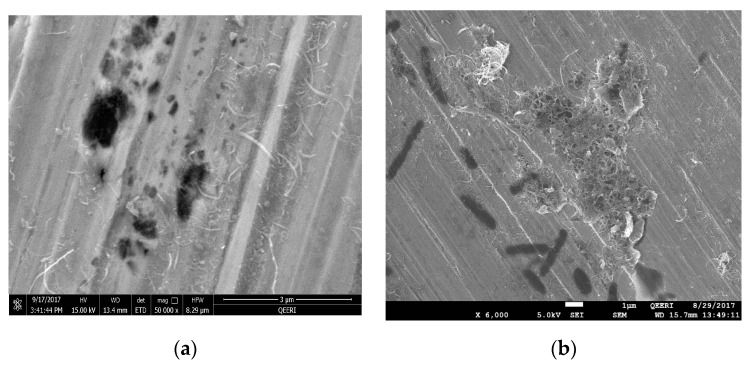

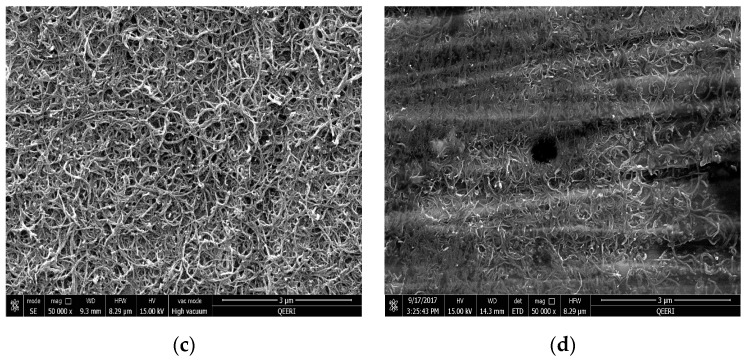
SEM images for 316L stainless steel samples after the potentiodynamic testing with different CNT loadings of CNT-water nanofluid, before washing with (**a**) 0.05, (**b**) 0.1, (**c**) 0.3 and (**d**) 0.5 wt%.

**Figure 9:**
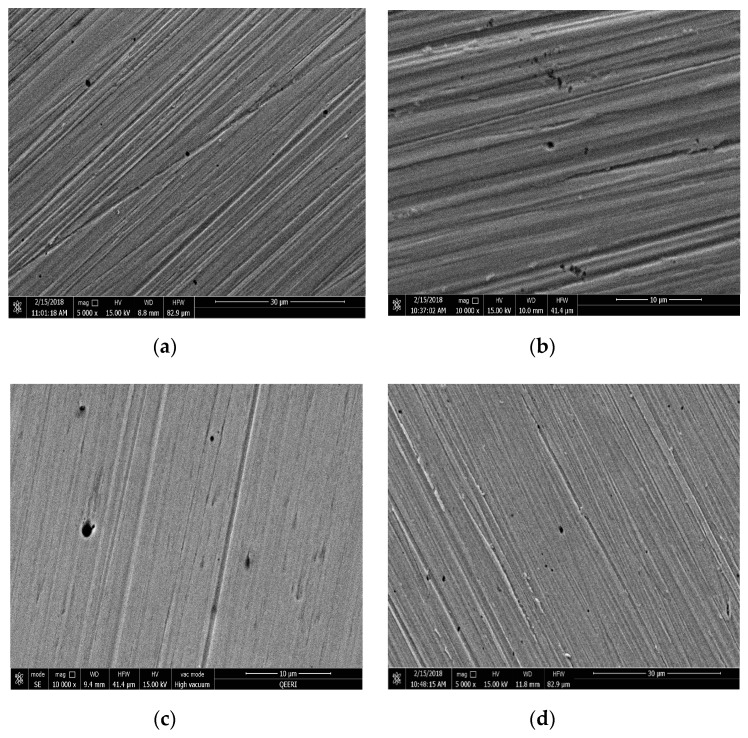
SEM images for 316L stainless steel samples after the potentiodynamic testing in different CNT loading of CNT-water nanofluid, after washing (**a**) 0.05, (**b**) 0.1, (**c**) 0.3 and (**d**) 0.5 wt%.

The surface morphology of the tested samples were observed using scanning electron microscopy (SEM) before and after washing with deionized water to inspect the effect of CNTs on the surface coating. SEM images can also show pits’ size, shape and density. The presence of CNTs was clearly observed on the surface of the metal, as shown in Figure 8a–d for samples tested in 0.05, 0.1, 0.3 and 0.5 wt% CNT-water nanofluid, respectively. The CNTs were randomly distributed on the surface of the metal, and sometimes accumulated around and inside the pits more than the rest of the surface, as seen in Figure 8b for the sample tested in 0.1 wt% CNT nanofluid. However, a uniform film coating of CNTs on the metal surface was observed on the surface of 0.3 and 0.5 wt% of CNT nanofluid samples, as it is shown in Figure 8c and Figure 9d, respectively. In addition, this coating was found to have a weak adhesion to the surface of the metal, as it was easily removed by rinsing the surface of the metal with deionized water. Such weak adhesion might have been due to the high agglomerations and aggregations of CNTs that reduced the attraction forces between CNTs and the metal surfaces. In Figure 9, washed samples tested in all CNT concentrations were viewed to have pits’ diameter in a range of a few microns, with a maximum diameter of 1.5 µm. However, the number of pits per surface area (pit density) was examined to be higher for the samples tested in higher CNTs loadings of 0.3 and 0.5 wt% CNTs.

**Figure 10:**
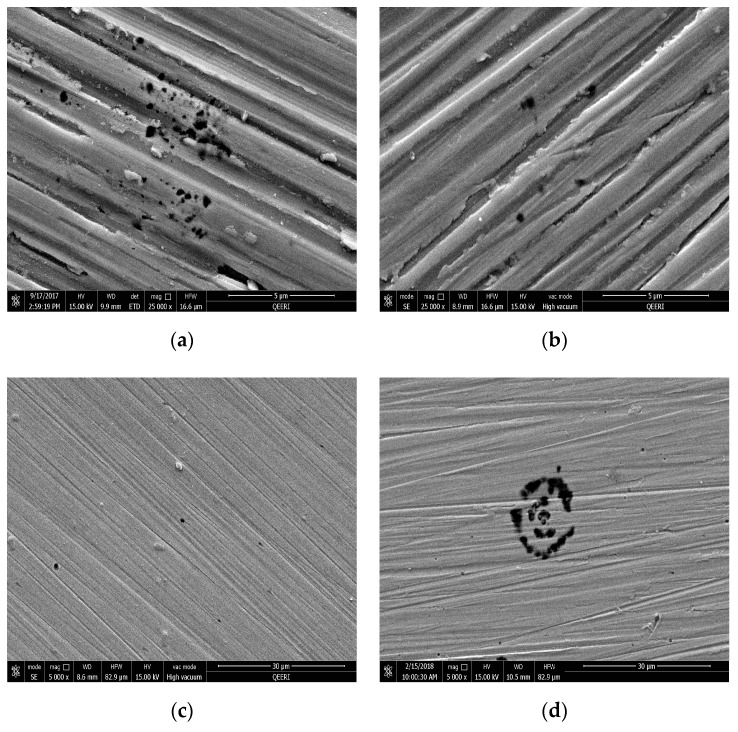
SEM images for 316L stainless steel samples after the potentiodynamic testing in different solutions: (**a**) tap water, (**b**) deionized water, (**c**) 1.5 wt% GA-water and (**d**) 3.5 wt% NaCl solution.

Figure 10 shows the surface SEM images of the metal samples tested in four different solutions: deionized water, tap water, 1.5 wt% GA-water and 3.5 wt% NaCl solutions. The diameter of the pits formed on the surface of metal in deionized and tap water samples were close to those formed in nanofluid samples. However, pit density formed in deionized and in tap water samples were much less than in the nanofluids. As for samples tested in GA-water solutions, all samples were shown to have higher pit densities and bigger pit diameters (around 1–10 µm) than the corresponding ones obtained for the CNT nanofluids. Bigger pits of around 30 µm diameters were formed with the corrosive NaCl solution. These images highlighted the higher corrosion rate happening in NaCl solution samples compared with all other samples. Samples tested in tap water and deionized water had the lowest pit densities, which is in agreement with lowest corrosion rates found there. The increased corrosion of samples tested in GA-solutions with respect to others exposed to all nanofluids was revealed too. Covering part of the exposed surface with CNTs increased the resistance of charge movements, delayed the propagation of these pits and, hence, decreased the corrosion rate, as can be seen from the smaller diameter of pits formed with nanofluids compared with those when tested in GA-only solutions. On the other hand, higher CNT concentrations exhibited higher pit densities, which indicates that CNTs accumulate in stagnant areas and introduce active anodic sites underneath CNTs, therefore increasing the chances to form pits there.

### 3.4. Proposed Corrosion Inhibition Mechanism with the Presence of CNTs

MWCNTs have the tubular shape and consist of carbon atoms that are connected to each other through covalent bonding. CNTs are suspended in solution using the anionic surfactant GA. The hydrophobic part of the GA (hydrocarbon chain) is attached to the hydrophobic surface of the CNTs, while the hydrophilic part of the surfactant (hydroxyl group) is connected to the polar molecules in water [82]. GA surfactant is a natural corrosion inhibitor in acidic and alkaline solutions [41,61]. It works as an inhibitor by getting adsorbed on the surface of the metal through its oxygen atom, and forcing the GA molecules to horizontally orient to the metal surface. Adsorption of the surfactant leads to an increase in surface blocking and protection efficiency, even at low surfactant concentrations [41,42]. Such inhibition effects of GA were not as noticeable in the samples tested in the GA-water solution as they were in deionized water, which had minimal amounts of ions and a very low corrosion rate.

The presence of CNTs and GA species influenced the passivation behavior of the metal, which was observed from the potentiodynamic scans. Furthermore, adsorbed CNTs affected surface energy and retarded both metal dissolution anodic reaction and hydrogen evolution reduction reaction, as indicated by E_corr_ shifts and Tafel slope values. However, from a thermodynamic perspective, it was revealed that CNTs had a dominant blocking effect, as they were physically attached to the surface of the metal by weak electrostatic forces. This physical adsorption followed the modified Langmuir adsorption isotherm, and there were interactions between the adsorbed CNTs and/or GA species. In addition, there were more than one of these species competing to occupy one active site of the surface. These results conclude that the mechanism of inhibition was mainly through physical blocking of CNTs/GA molecules. Surface blocking hinders corrosion reactions by increasing mass diffusion resistance and by providing obstructions that prevent the solution ions from reaching the metal’s surface.

However, at higher concentrations, surface energy might dominate the blocking effect and increase corrosion of the steel surface. This might be due to the formation of active anodic sites underneath the CNTs, which were shown to cover the metal’s surface non-uniformly. The interfacial area between CNTs and the metal surface is a stagnant area, which causes ion accumulations and lowers the pH site. In addition, uncovered area have electrons liberated from metal dissolution that tend to react with adsorbed oxygen and water molecules in the solution, creating cathodic regions there. This oxygen reduction reaction tends to increase the site potential and create difference in the potential between covered and uncovered areas. Therefore, active anodic sites are created under covered areas, which increases the possibility of pit initiations. At the same time, CNTs cover part of the surface, which increases diffusion resistance against the surrounding ions trying to reach the surface of the metal, and retards corrosion. At higher CNT concentrations, the surface energy effect increases and dominates the blocking effect. This will cause the metal to dissolute.

## 4. Conclusions

The corrosion properties of the 316L SS samples were tested under different types of solutions including CNT-water nanofluids, GA-water, deionized water, tap water and 3.5 wt% NaCl solutions. Samples tested in CNT nanofluids were shown to have relatively low corrosion rates and were comparable to steel tested in tap water and in deionized water. In addition, lower corrosion rates were obtained in all CNT nanofluids than in to those for GA-deionized water solutions of equivalent GA concentrations. A lowest corrosion rate of 6.43 mmpy (milli mil per year) was obtained when testing was conducted in 0.1 wt% CNT nanofluid at room temperature. These test conditions achieved the highest inhibition efficiency of 61%. The inhibition performance of CNTs was due to the physical adsorption of CNTs on the surface of the metal. CNTs formed an external layer that worked as a barrier and prevented the solution ions from attacking the metal’s surface. From a thermodynamic perspective, Gibbs free energy (ΔG°_ads_) value was −15.73 kJ/mol, indicating that CNTs and GA were physically adsorbed on the surface of the metal. Furthermore, the adsorption process followed the modified Langmuir type I adsorption isotherm, where there are interactions between adsorbed species, and there is more than one CNT and/or GA molecule adsorbed at a particular active site.

The inhibition efficiency of CNTs was found to decrease with increasing CNT concentrations. At higher CNT concentrations, a layer of CNTs formed on the surface of the metal in an uneven and a non-uniform distribution. This could cause ion accumulation on stagnant regions that will become active anodic sites and stimulate corrosion reactions. SEM images showed the biggest pit diameters and highest densities for the steel tested in 0.5 wt% CNT-water nanofluids.

## Figures and Tables

**Table 1:** Specifications of multiwalled carbon nanotubes (MWCNTs) used in the present research.

Outer Diameter	Inside Diameter	Purity	Length	Specific Surface Area	Electrical Conductivity	Bulk Density	True Density
20–30 nm	5–10 nm	>95%	10–30 µm	110 m^2^/g	>100 S/cm	0.28 g/cm^3^	~2.1 g/cm^3^

**Table 2:** Composition of tap water used in testing [53].

Constituent	Bromide (Br^−^)	Bicarbonate (HCO_3_^−^)	Carbonate (CO_3_^−^)	Fluoride (Fl^−^)	Chloride (Cl^−^)	Nitrate (NO_3_^−^)
Amount (mg/L)	0.3	76.9	<1.0	0.1	16.3	<0.01
Constituent	Total Organic Carbon (TOC)	Total Nitrogen	Sulphate (SO_4_^−2^)	Calcium (Ca)	Magnesium (Mg)	Total Dissolved Solids (TDS)
Amount (mg/L)	0.3	0	<2.0	25	1.2	92

**Table 3:** Composition of 316L stainless steel purchased from the company Metals Samples (weight percentages).

Fe	Cr	Ni	Mo	C	Mn	Si	Cu	Co	Others
68.28	16.65	10.1	2.03	0.019	1.48	0.48	0.46	0.37	0.14

**Table 4:** Corrosion potential values for 316L stainless steel tested in different solutions at room temperature.

Solutions in Deionized Water (Percentages in wt%)	E_corr_ (mV_SCE_)	ΔE_corr_ (mV_SCE_)
0.05% CNT + 0.15% GA	−49	27
0.1% CNT + 0.3% GA	−38	39
0.3% CNT + 0.9% GA	−130	59
0.5% CNT + 1.5% GA	−108	41
0.15% GA	−76	-
0.3% GA	−77	-
0.9% GA	−189	-
1.5% GA	−149	-

**Table 5:** Corrosion parameters for 316L stainless steel tested in different solutions at room temperature.

Solution (Compositions in wt%)	βa (V/decade)	βc (V/decade)	Epit (V_SCE_)	i_corr_ (µA/cm^2^)	Rp (kohm)	Corrosion Rate (mmpy)
**Tap water**	0.1038	0.0894	0.963	0.0145	4649	4.53
**3.5% NaCl**	0.2641	0.1527	0.187	0.1488	839	44.34
**Deionized Water**	0.4668	0.0762	1.100	0.0106	7994	3.44
**0.05% CNT + 0.15% GA**	0.2729	0.1375	1.147	0.0244	4984	7.47
**0.1% CNT + 0.3% GA**	0.2644	0.1384	1.128	0.0199	5950	6.43
**0.3% CNT + 0.9% GA**	0.3917	0.1266	0.957	0.0236	5503	7.12
**0.5% CNT + 1.5% GA**	0.2973	0.1194	0.882	0.0304	3777	9.45
**0.15% GA**	0.8012	0.1302	1.061	0.0556	2634	17.31
**0.3% GA**	0.4370	0.1617	1.050	0.0496	2853	16.30
**0.9% GA**	0.5350	0.1320	0.961	0.0367	3667	11.40
**1.5% GA**	0.3749	0.1621	1.059	0.0410	3792	12.45

**Table 6:** Inhibition efficiency (*IE*%) and surface coverage (*θ*) of 316L stainless steel samples tested in different CNT nanofluids (mmpy is milli mil per year and µm/yr is micrometer per year).

Solution (Compositions in wt%)	CNTs or GA Concentration (*C* in g/L)	Corrosion Rate (mmpy)	Corrosion Rate (µm/yr)	*IE* (%)	Surface Coverage (*θ*)
0.05% CNT + 0.15% GA	0.05	7.47	0.190	57	0.57
0.1% CNT + 0.3% GA	1	6.43	0.163	61	0.61
0.3% CNT + 0.9% GA	3	7.12	0.181	38	0.38
0.5% CNT + 1.5% GA	5	9.45	0.240	24	0.24
0.15% GA	1.5	17.31	0.440	-	-
0.3% GA	3.0	16.30	0.414	-	-
0.9% GA	9.0	11.40	0.290	-	-
1.5% GA	15	12.45	0.316	-	-
